# Real-Time Tracking by Double Templates Matching Based on Timed Motion History Image with HSV Feature

**DOI:** 10.1155/2014/793769

**Published:** 2014-01-22

**Authors:** Zhiyong Li, Pengfei Li, Xiaoping Yu, Mervat Hashem

**Affiliations:** College of Information Science and Engineering, Hunan University, Changsha 410082, China

## Abstract

It is a challenge to represent the target appearance model for moving object tracking under complex environment. This study presents a novel method with appearance model described by double templates based on timed motion history image with HSV color histogram feature (tMHI-HSV). The main components include offline template and online template initialization, tMHI-HSV-based candidate patches feature histograms calculation, double templates matching (DTM) for object location, and templates updating. Firstly, we initialize the target object region and calculate its HSV color histogram feature as offline template and online template. Secondly, the tMHI-HSV is used to segment the motion region and calculate these candidate object patches' color histograms to represent their appearance models. Finally, we utilize the DTM method to trace the target and update the offline template and online template real-timely. The experimental results show that the proposed method can efficiently handle the scale variation and pose change of the rigid and nonrigid objects, even in illumination change and occlusion visual environment.

## 1. Introduction

As a hot research topic in computer vision, moving object tracking has numerous applications such as video surveillance, visual navigation, and human-computer interaction. However, it remains a tough problem to track the target under complex environment due to the scale variation, pose change, illumination change, occlusion, and real-time processing requirement. To overcome these difficulties, many object tracking methods have been proposed in recent years [1].

Traditional tracking algorithms such as mean-shift [2] and particle filter [3] are well developed in the past few years. Many extensions have emerged. Recently, Leichter [4] proposed a tracker using cross-bin metrics based on mean-shift, which is a simple and efficient method. Mei and Ling [5] treated tracking problem as a sparse approximation problem in a particle filter framework. As we know, an important aspect that determines the performance of the tracking algorithm is the object's appearance model. However, these traditional methods cannot represent an enjoyable appearance model, which results in poor jobs on handling the scale variation, pose change, and some complicated visual environment such as occlusion and illumination change.

Kass et al. [6] proposed snakes models to deal with the pose change, but the tracking process is a solution optimization process with high computational complexity. Level sets [7], which represent a contour using a signed distance map, were used to draw accurate active contour for tracking. The condensation algorithm [8] used the B-spline curve to parameterize the contour and particle filtering method for tracking. Ross et al. [9] used an incremental subspace model to adapt appearance changes. However, these methods are undesirable because a drift problem may appear once the target object's appearance changes significantly during the tracking process.

Online learning has spawned the approach of tracking by detection, which treats the tracking as a binary classification problem. Collins et al. [10] utilized online feature selection to trace the target. Avidan [11] proposed ensemble tracking method that extended mean-shift with adaBoost. Grabner and Bischof [12] proposed an online boosting tracker which built a feature selection framework for tracking. More recently, Santner et al. [13] built a sophisticated tracking system called PROST which is robust. Zhang et al. [14] proposed a compressive tracking algorithm that used compressing sensing theories to extract features for real time tracking. Nevertheless, because the appearance model and background must be learned at frame rate and the training data for classifier is numerous, thus, these tracking methods are not efficient for real-time tracking. Meanwhile, it also often leads to tracking drift problem since the appearance model is updated with noise and a few negative examples.

In this paper, we propose a novel method in which the appearance model is described by object templates based on tMHI-HSV. Firstly, a novel moving object segmentation and modeling method named tMHI-HSV are proposed, which is robust and efficient to select and describe candidate object patches. Moreover, the DTM strategy is applied to ensure the accuracy and robustness of the tracking. The online template is dynamically updated in real time to adapt the change of target appearance, and the offline template is updated to deal with the overfitting problem that is caused by the fast changes of environment.

The rest of this paper is organized as follows. We introduce the motion history (MHI) method in Section 2.  Section 3 presents the DTM tracking method based on tMHI-HSV. Experimental results are shown and discussed in Section 4. Finally Section 5 concludes the paper.

## 2. MHI Method

### 2.1. Motion History Image

The motion history image (MHI) method is an approach based on template matching. The MHI with current motion pixels of images (>2 frames) updated using a timestamp can provide abundant motion information of a moving object.

Ahad et al. [15] had made a survey on MHI method and its applications. As an impressive method for motion representation, MHI is a standard technique in computer vision. Bobick and Davis [16] utilized MHI as temporal templates to recognize the human movement. Zhaozheng and Collins [17] proposed a forward-backward MHI method to locate the moving object in thermal imagery. Lin et al. [18] used MHI method to segment motion region that was more robust than segmenting objects in one frame for tracking moving object. Davis et al. [19] separated human motion patterns from the noise categories by representing the moving object with a minimum spatial size and temporal length based on MHI approach. Using MHI method to represent the motion appearance is simple and effective. An MHI image is computed as follows.

Let *f* = [*f*_1_(*x*, *y*),…, *f*_*i*−1_(*x*, *y*), *f*_*i*_(*x*, *y*),…, *f*_*n*_(*x*, *y*)] denote a video frame stream. The absolute value of frame differencing result *D*_*t*_(*x*, *y*) is computed as

(1) $$\begin{array}{c} D_{t}\left(x,y\right)=\;\left|f_{i}\left(x,y\right)-f_{i-1}\left(x,y\right)\right|, \end{array}$$

where *f*_*i*_(*x*, *y*) and *f*_*i*−1_(*x*, *y*) are two adjacent frames of current time *t* from video frames. The update function *B*_*t*_(*x*, *y*) is defined by *D*_*t*_(*x*, *y*) based on a threshold *σ*. Consider

(2) $$\begin{array}{c} B_{t}\left(x,y\right)=\{\begin{array}{cc} 1 & \text{if}\;D_{t}\left(x,y\right)>\sigma \\ 0 & \text{otherwise}, \end{array} \end{array}$$

then, the MHI_*t*_(*x*, *y*) is computed according to the update function *B*_*t*_(*x*, *y*):

(3) $$\begin{array}{c} {\text{MHI}}_{t}\left(x,y\right)=\{\begin{array}{cc} t & \text{if}\;B_{t}\left(x,y\right)=1\\ {\text{MHI}}_{t-1}\left(x,y\right) & \text{otherwise}. \end{array} \end{array}$$

Since the motion information is preserved in the MHI, it can represent the motion object in a continuous way. Thus, the MHI template is insensitive to some interference, like illumination change and occlusion. These advantages make MHI method suitable for motion analysis in challenging scenarios.

### 2.2. Timed MHI Generation

**Figure 1:**
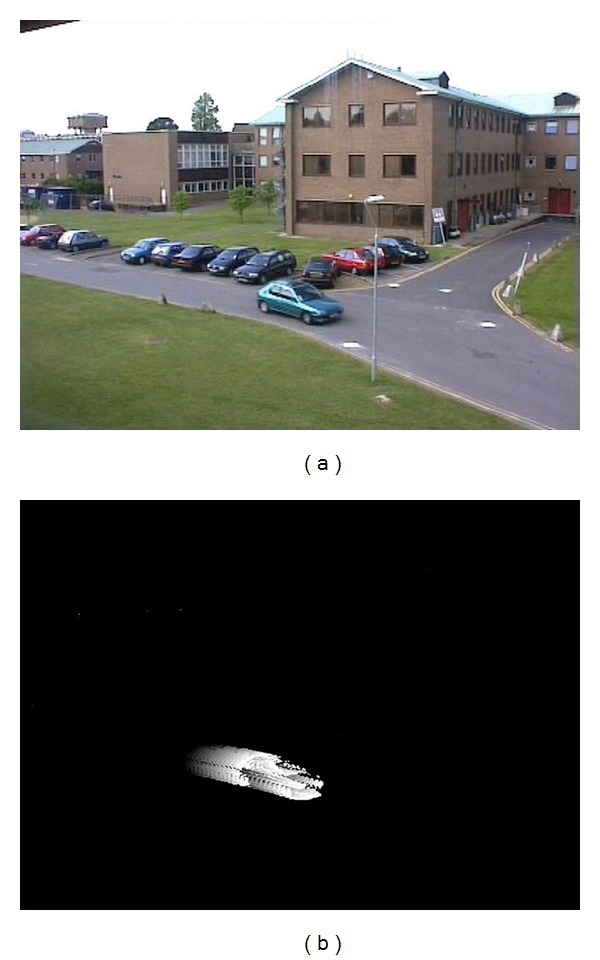
(a) The original image of the car. (b) The tMHI of the car. Here, *σ* = 20 and *δ* = 10 s.

Timed motion history image (tMHI) is a smart motion segmentation method [20] that is extended from MHI. A history of temporal changes is kept at each pixel location and then decays over time. The tMHI utilizes a floating-point MHI where new silhouette values are represented with floating-point timestamp. Meanwhile, the tMHI is updated with the timestamp of current system. The more recent pixels of the moving object have higher intensity. The tMHI image is computed as

(4) $$\begin{array}{c} \text{t}{\text{MHI}}_{\delta }\left(x,y\right)=\{\begin{array}{cc} \tau & \text{if}\;\text{current}\;\text{silhouette}\;\text{at}\;\left(x,y\right)\\ 0 & \text{else}\;\text{if}\;\text{t}{\text{MHI}}_{\delta }\left(x,y\right)<\left(\tau -\delta \right), \end{array} \end{array}$$

where *τ* is the current timestamp and *δ* is the decay parameter that determines the motion length.  Figure 1 shows the tMHI of a car. From Figure 1, we can conclude that the tMHI provides coherent motion information to represent the motion trail of moving object over time.

## 3. tMHI-HSV-Based DTM Tracking Method

**Figure 2:**
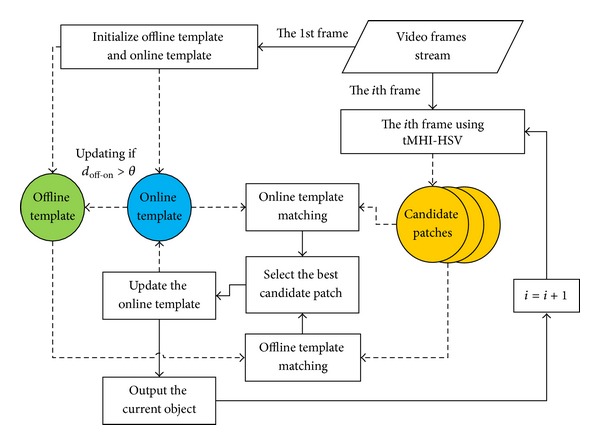
Block diagram of the proposed method.

This section provides a detailed description of the proposed method. The tracking algorithm starts with the work by initializing the tracking window and gets the same offline template and online template in the first frame by user specifying the object region. Next, for each frame of the next video frames stream, some potential candidate moving object patches are screened out by using tMHI-HSV. The *Bhattacharyya distance* measure is used to measure the similarities between each candidate patch and online template, as well as offline template. The patch with the minimum *Bhattacharyya distance* is chosen as the best candidate object in current frame, and its location position and silhouette are outputted as the current object spatial information. Furthermore, the online template is updated by the current frame object patch. Meanwhile, the difference of the offline template and the newest online template is analyzed; the offline template may be updated if the difference is too large. So the tracking and updating cycle will be continued for the whole video steam; the block diagram is shown in Figure 2.

### 3.1. tMHI-HSV Representation

The tMHI method is used to detect the moving region and segment the moving objects to obtain MHI silhouettes. Before the segmentation, a median filter is employed to eliminate salt and pepper noise. Then, the morphological operation is adopted to remove the target's discontinuous hollow. A threshold Δ_MinSize_, which is determined by the target's spatial size, is set to find the potential candidate MHI external rectangle silhouettes. The MHI silhouettes whose sizes are larger than Δ_MinSize_ are screened out as the candidate MHI silhouette sets **C** as follows:

(5) $$\begin{array}{c} C=\left\{c_{1},c_{2},\ldots ,c_{m}\;|\;S_{c_{i}}\geq \Delta_{\text{MinSize}},\;i=1,2,\ldots ,m\right\}. \end{array}$$

Here, *S*_*c*_ denotes spatial size of the silhouettes *c*, and *m* is the total number of silhouettes. Generally *S*_*c*_ = *r*_width_ × *r*_height_; *r*_width_ and *r*_height_ are the width and height of the rectangle silhouette *c* in pixels, respectively. This processing can help to remove noise pixels from camera jitter and small motion such as shaking leaves.

However, the MHI silhouette cannot describe the whole of the candidate patch information except the spatial motion feature. Meanwhile, it is lack of robustness and accuracy to track the target just according to the spatial size and temporal length. Hence, one kind of global feature, color feature, is employed and fused with tMHI to exactly represent these candidate patches' specialties. Compared to the RGB system, HSV system describes more accurately than RGB color system on perception links and remains computationally simple. Therefore, the HSV system is chosen to represent those patches' color feature.

Thus, each candidate patch *c* can be described by the HSV color histogram **H** of its tMHI region. **H** = {*h*(*b*) | *b* = 1,2,…, *q*}, which is named tMHI-HSV feature in this paper, and *h*(*b*) denotes the number of pixels that belong to the color bin *b*. The color histogram **H** can be computed as follows:

(6) H={h(b)}={∑i=1NQ(pi,b)}, b=1,2,…q,Q(pi,b)={1if  pi  belong  to  color  bin  b,0otherwise,

where *b* is one of the color bins, *q* is the total number of color bins, *N* is the number of pixels in this candidate patch *c*, and *p*_*i*_ is the color value of the *i*th pixel. Rather than only the spatial feature, the color feature is used for modeling the candidate patches and templates.

### 3.2. Double Template Matching

During the whole tracking process, the general method [21] initializes the first frame to delimit the tracking window as the template region. However, a series of changes such as scale variation and pose change may occur on the target during the tracking process. In addition, changes in the visual environment can cause interference. The appearance change and circumstance disturbing always lead to a drift problem and even tracking lost, because the original template cannot accurately represent the appearance model of the current target. Therefore, updating template online in real time during tracking is very crucial. However, an over-fitting problem would occur for online template matching when the visual environment and appearance change rapidly and noisily. For example, the illumination changes sparklingly. For the sake of avoiding the online template over-fitting learning problem, one stable offline template is reserved as the second matching template. The patch can be chosen as the best candidate current object if it has the minimum similarity degree to the online template and offline template. This method is called the double template matching (DTM) here.

Generally, the target's appearance changes only a little between two adjacent frames *f*_*i*−1_ and *f*_*i*_. It is a reasonable way that uses the object patch in the previous frame as the online template to trace the target in the next frame. Here, the *Bhattacharyya distance* of tMHI-HSV color histogram is used to measure the similarity between the online templates *T*_Online_ and the candidate object patch *c*_*i*_.

According to the definition of *Bhattacharyya distance* based on two vectors **p** = {*p*_1_, *p*_2_,…, *p*_*k*_} and **q** = {*q*_1_, *q*_2_,…, *q*_*k*_}, we have

(7) $$\begin{array}{c} d_{Bhattacharyya}\left(p,q\right)=\sqrt{1-\frac{\sum_{k}\sqrt{p_{k}\times q_{k}}}{\sum_{k}p_{k}\times \sum_{k}q_{k}}}. \end{array}$$

Thus, the online template matching and the offline template matching solve this optimization problem as follows:

(8) $$\begin{array}{c} j_{\text{Online}}=\underset{i}{\arg\;\min}\left\{d_{Bhattacharyya}\left(c_{i},T_{\text{Online}}\right)\right\},\\ j_{\text{Offline}}=\underset{i}{\arg\;\min}\left\{d_{Bhattacharyya}\left(c_{i},T_{\text{Offline}}\right)\right\}. \end{array}$$

Moreover, the best candidate patch *c*_Best_ can be chosen from the two minimum *Bhattacharyya distance* patches as follows:

(9) cBest ={cj ∣ j=arg min⁡jOnline,jOffline{dBhattacharyya(cjOnline,TOnline),dBhattacharyya(cjOffline,TOffline)}}.

So, *c*_Best_ can be the best candidate object in the current frame to be outputted. Here, double template matching is adopted to avoid online template over-fitting problem. When the online template has overlearned some noise change from the last several frames, the tracking drift will occur. This means that the tracking method will match the current object to wrong patch. The proposed method can ameliorate this phenomenon because an offline template has been adopted at the same time. The offline template is a more stable template in this situation, because it learns less from the object appearance than online template.

### 3.3. Template Updating

In order to keep the tracking accuracy, templates updating is the key procedure in this proposed method. Here, the current best candidate object patch *c*_Best_ is as the newest online template of the next frame as follows:

(10) $$\begin{array}{c} T_{\text{Online}}=c_{\text{Best}}. \end{array}$$

Meanwhile, an over-fitting problem caused by online template matching may occur when the visual environment changes rapidly and randomly. For example, the illumination suddenly changes sparklingly or the object goes through some obstruct continuously and quickly. To avoid this problem, a threshold *θ*, which is set to 0.3 by experimental picking in this paper, is used to evaluate whether the offline template needs to be updated or not. Compared to the current online template *T*_Online_, the offline template can be updated as follows:

(11) $$\begin{array}{c} d_{\text{Off-On}}=d_{Bhattacharyya}\left(T_{\text{Offline}},T_{\text{Online}}\right),\\ T_{\text{Offline}}=\{\begin{array}{cc} T_{\text{Online}} & \text{if}\;d_{\text{Off-On}}>\theta \\ T_{\text{Offline}} & \text{otherwise}. \end{array} \end{array}$$

As above, the offline template is updated only when it differs much from the online template. So this strategy guarantees that our method is more robust under the frequently changing environment.

### 3.4. Algorithm Pseudocode

This proposed method's pseudocode is shown in Algorithm 1.

## 4. Experimental Results and Analysis

The proposed algorithm is evaluated against scale variation, pose change, occlusion, and illumination change. Our tracker is compared with two state-of-art algorithms, the online boosting tracker (OBT) [12], and compressive tracking (CT) [14] methods. The *red*, *yellow*, and *green rectangle windows* are used to mark the target tracked by our method, OBT and CT, respectively. The source codes, which are provided by the authors, are used for the comparison purpose.

The parameters of the video sequences are displayed in Table 1, and the initialization algorithm parameters are shown in Table 2. The binary threshold *σ* is set from 20 to 30 and the offline template updating threshold *θ* is set as 0.3, whereas Δ_MinSize_ and *δ* can be set depending on the practical application as in Table 2. We run the experiments on a PC with Intel Pentium 2.70 GHz CPU and 2 GB RAM. The tracking rates frames per second (FPS) obtained by using the proposed method can reach 16FPS, 21FPS, and 62FPS corresponding to the video sizes 768 × 576, 640 × 480, and 320 × 240, respectively, which demonstrates that our method is well positioned to meet the real-time requirement.

### 4.1. Qualitative Evaluation

**Figure 3:**
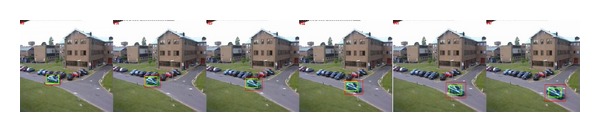
Tracking results of *PETS2000* sequence of *blue car*. Frames 5, 14, 23, 32, 41, and 47 are displayed.

**Figure 4:**
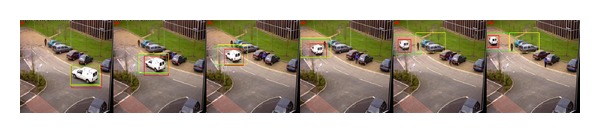
Tracking results of *PETS2000* sequence of *white car*. Frames 5, 25, 45, 65, 85, and 105 are displayed.

*Scale Variation*. We utilize the *PETS2000* sequences of cars to test the performance of OBT, CT, and our method in handling the scale variation. The scale of the car changes from small to big in the *blue car* sequence and it changes from big to small with rotation in the *white car* sequence. As we can see from the tracking results shown in Figure 3, the three trackers are all able to trace the car. However, we can see that CT and our method perform well, while the OBT method fails when the scale of the white car becomes smaller as in Figure 4. In addition, the tracking windows of OBT and CT cannot adaptively change with the scale variation of the car. 

**Figure 5:**
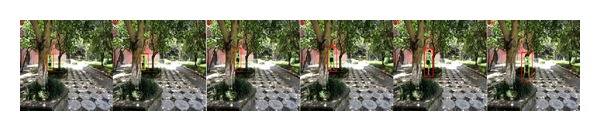
Tracking results of *girl pose* sequence. Frames 5, 25, 45, 65, 85, and 105 are displayed.

**Figure 6:**
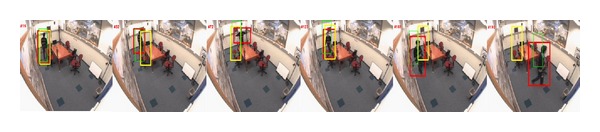
Tracking results of *intelligent room* sequence. Frames 16, 32, 72, 123, 161, and 193 are displayed.

*Pose Change*. We use the *girl pose* sequence captured by ourselves and *intelligent room* sequence to evaluate the trackers' abilities in handling pose change problem. Some tracking results are illustrated in Figures 5 and 6. From Figure 5, we note that our tracker outperforms OBT and CT methods. No matter how the girl moves, our tracker is satisfied while the others are not. As for the *intelligent room* sequence, the man has a pose change. Into the bargain, his scale changes a lot. Both of the OBT and CT methods produce drift problems. OBT tracker even lost the target, whereas our method utilizes tMHI and an online template is updated in real time to adapt the target's pose change. Thus, we obtain better results. 

**Figure 7:**
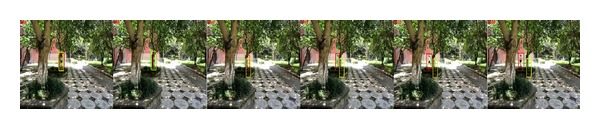
Tracking results of *girl occlusion*. Frames 83, 93, 103, 113, 123, and 133 are displayed.

*Occlusion*. The *girl occlusion* sequence is used to test the performance of our tracker when the target is under heavy or even complete occlusion. Part of the tracking results is presented in Figure 7. From 83th and 93th frames, we note that the three trackers all succeed in tracking the target when the partial occlusion occurs. However, the OBT and CT methods fail to track the target when the occlusion is heavy or even complete while our method performs well. The tMHI and DTM make contribution to handle this thorny problem. A detector is included in tMHI, and the motion trail of the target can learn from it. We cannot learn the online template to describe the target, whereas the offline template is valuable when the complete occlusion happens. Therefore, a satisfied result is produced by our method. 

**Figure 8:**
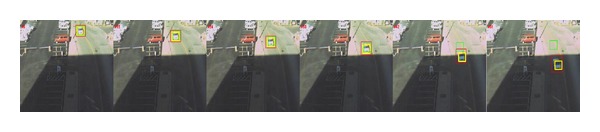
Tracking results of* car sequence*. Frames 6, 15, 24, 33, 42, and 51 are displayed.

*Illumination Change*. For the tracking results of *car sequence* shown in Figure 8, the illumination changes significantly. Our method and OBT perform well in this situation, while the CT tracker fails to track the target when the car moves from bright region to dark region. Our online tracking template is updated in real time to adjust to the target's appearance variation. In addition, the tMHI-HSV feature is insensitive to illumination change. These advantages make our approach robust to illumination change.

### 4.2. Quantitative Evaluation

**Figure 9:**
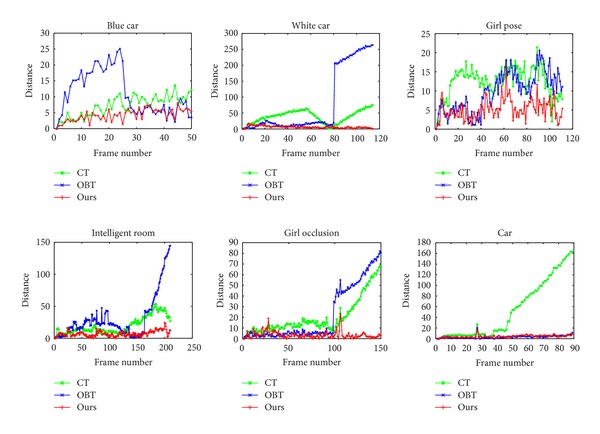
Results of CLE.

In addition to the qualitative evaluation, the success rate (SR) and center location error (CLE) measured with manually labeled ground truth data are used for the quantitative evaluation. The score of SR is defined as

(12) $$\begin{array}{c} \text{score}=\frac{\text{area}\left({\text{ROI}}_{\text{T}}\cap {\text{ROI}}_{\text{G}}\right)}{\text{area}\left({\text{ROI}}_{\text{T}}\cup {\text{ROI}}_{\text{G}}\right)}, \end{array}$$

where ROI_T_ is the tracking bounding box and ROI_G_ is the ground truth tracking box. We consider the tracking result as a success if the score is larger than 0.5 in one frame. The SRs presented in Table 3 demonstrate that our method achieves the best result or the second best result. The distance (pixel) between the center location of the tracked target and the ground truth is used to measure the CLE. The results of CLE shown in Figure 9 illustrate that our method outperforms the other two methods. For the *blue car* sequence, the OBT performs slightly worse, although the three trackers have low CLEs. The OBT fails to track the car after the 80th frame in the *white car* sequence. CT and our method succeed in tracking the white car, whereas our method produces very few CLEs. The distances calculated from *girl pose* sequence are all lower than 25, yet our method shows a better result on the whole sequence. In terms of *intelligent room* sequence, OBT lost the target after the 160th frame and the distances produced by CT are larger than that generated through our method. As to the *girl occlusion* sequence, both OBT and CT are unable to track the girl when she is under heavy or even complete occlusion. However, our method maintains the performance in such scenario. From the results of *car* sequence shown in Figure 9, we note that the CT cannot handle the illumination change problem, while OBT and our method perform well.

## 5. Conclusions and Future Work

A real-time tracking method with object tMHI-HSV appearance model and double templates (offline and online) matching has been presented. The initial offline template and online template are generated using the original shape and HSV color histogram feature of the target. On the current frame's candidate patches, we utilize the tMHI to segment them. Rather than the spatial pixel template, the HSV color feature is used in our method, which reduces the computational complexity and increases the robustness. These advantages make the motion appearance expression simple and effective. Double template matching is adopted to exactly determine the target location. Meanwhile, the online template is updated real-timely, and offline template is updated as needed. We evaluate our method in six scenarios. The tracking rates illustrate that the proposed algorithm meets the real-time requirement, and the comparative experiments demonstrate that our method outperforms the other two schemes in terms of accuracy and robustness.

For future work, we will extend the proposed method and apply it on a moving camera rather than a fixed one, as we used in our experiment. Considering that features are very important for moving object tracking, other features except HSV color histogram can be used.

## Figures and Tables

**Algorithm 1:**
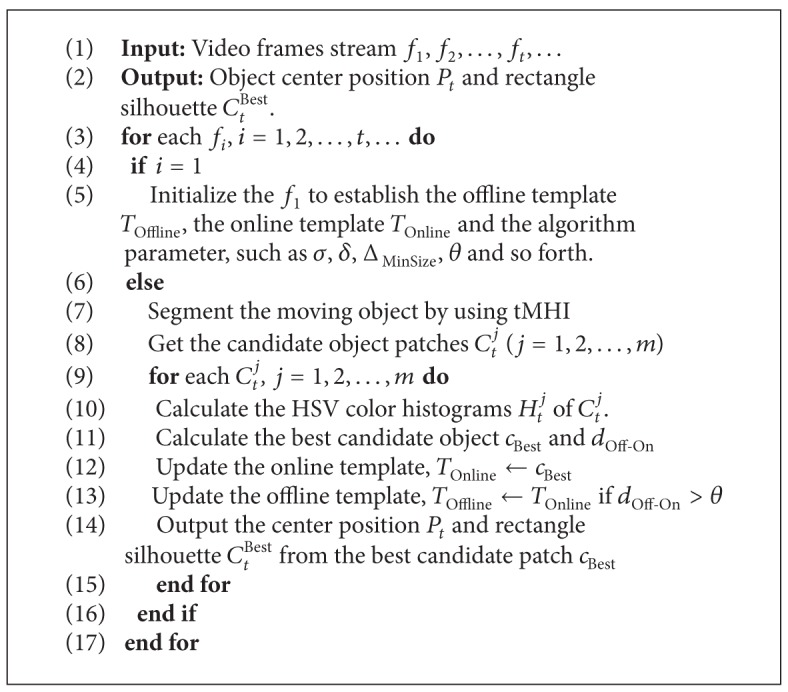
tMHI-HSV-based DTM tracking.

**Table 1:** Video parameters.

Video sequence	Video parameters		
	Frame size/pixel	Total frames	Target
Blue car	768 × 576	50	Blue car
White car	768 × 576	114	White car
Girl pose	640 × 480	111	Girl
Intelligent room	320 × 240	210	Man
Girl occlusion	640 × 480	150	Girl
Car	320 × 240	89	Car

**Table 2:** Algorithm parameters.

Video sequence	Algorithm parameters		
	*σ*	*δ*	Δ_MinSize_
Blue car	20	0.2	500
White car	20	0.7	500
Girl pose	25	1.5	1000
Intelligent room	25	0.8	1000
Girl occlusion	30	1.5	1000
Car	20	0.3	500

**Table 3:** Success rate (%).

Video sequence	Method		
	CT	OBT	Ours
Blue car	80	76	98
White car	14	13	90
Girl pose	69	70	87
Intelligent room	44	29	90
Girl occlusion	70	64	95
Car	38	88	87

Average SR	52	56	91
